# Exercise training attenuates renovascular hypertension partly via RAS- ROS- glutamate pathway in the hypothalamic paraventricular nucleus

**DOI:** 10.1038/srep37467

**Published:** 2016-11-24

**Authors:** Yan Zhang, Xiao-Jing Yu, Wen-Sheng Chen, Hong-Li Gao, Kai-Li Liu, Xiao-Lian Shi, Xiao-Yan Fan, Lin-Lin Jia, Wei Cui, Guo-Qing Zhu, Jin-Jun Liu, Yu-Ming Kang

**Affiliations:** 1 Department of Physiology and Pathophysiology, Key Laboratory of Environment and Genes Related to Diseases, Xi’an Jiaotong University School of Basic Medical Sciences, Xi’an Jiaotong University Health Science Center, Xi’an Jiaotong University Cardiovascular Research Center, Xi’an, 710061, China; 2 Department of Cardiovascular Surgery, Xijing Hospital, Fourth Military Medical University, Xi’an, 710032, China; 3 Department of Pharmacology, School of Basic Medical Sciences, Xi’an Jiaotong University Health Science Center, Xi’an, 710061, China; 4 Department of Endocrinology and Metabolism, The First Affiliated Hospital of Xi’an Jiaotong University, Xi’an Jiaotong University Health Science Center, Xi’an, 710061, China; 5 Key Laboratory of Cardiovascular Disease and Molecular Intervention, Department of Physiology, Nanjing Medical University, Nanjing, 210029, China

## Abstract

Exercise training (ExT) has been reported to benefit hypertension; however, the exact mechanisms involved are unclear. We hypothesized that ExT attenuates hypertension, in part, through the renin-angiotensin system (RAS), reactive oxygen species (ROS), and glutamate in the paraventricular nucleus (PVN). Two-kidney, one-clip (2K1C) renovascular hypertensive rats were assigned to sedentary (Sed) or treadmill running groups for eight weeks. Dizocilpine (MK801), a glutamate receptor blocker, or losartan (Los), an angiotensin II type1 receptor (AT1-R) blocker, were microinjected into the PVN at the end of the experiment. We found that 2K1C rats had higher mean arterial pressure (MAP) and renal sympathetic nerve activity (RSNA). These rats also had excessive oxidative stress and overactivated RAS in PVN. Eight weeks of ExT significantly decreased MAP and RSNA in 2K1C hypertensive rats. ExT inhibited angiotensin-converting enzyme (ACE), AT1-R, and glutamate in the PVN, and angiotensin II (ANG II) in the plasma. Moreover, ExT attenuated ROS by augmenting copper/zinc superoxide dismutase (Cu/Zn-SOD) and decreasing p^47phox^ and gp^91phox^ in the PVN. MK801or Los significantly decreased blood pressure in rats. Together, these findings suggest that the beneficial effects of ExT on renovascular hypertension may be, in part, through the RAS-ROS-glutamate pathway in the PVN.

Recent studies indicate that exercise training (ExT) is beneficial to hypertension in patients and animals^1^,^2^. The favourable effect of exercise training is due, in part, to decreased sympathetic activity and improved autonomic function^3^,^4^. Evidence suggests that ExT is associated with neuronal plasticity in the brain, which regulates blood pressure^5^,^6^. The role of ExT on glutamate within the rostral ventrolateral medulla (RVLM) and the associated improvement in sympathetic outflow has been extensively demonstrated in hypertension^7^. In other studies, ExT restores the balance between excitatory and inhibitory neurotransmitters and between pro- and anti-inflammatory cytokines, attenuates total reactive oxygen species (ROS) and superoxide production, and increases antioxidants within the paraventricular nucleus (PVN) of spontaneously hypertensive rats (SHR)^8^,^9^.

The rennin-angiotensin system (RAS) is involved in the pathophysiology of renovascular hypertension^10^,^11^,^12^. It has been reported that 2K1C (two-kidney, one-clip) renovascular hypertensive rats show a significant increase in mRNA and protein expression of the angiotensin II type 1 receptor (AT1-R) and angiotensin-converting enzyme (ACE) within the PVN^13^. Recent studies indicate that RAS in the PVN exerts its actions mainly via interaction with AT1-R and ACE, thereby contributing to sympathoexcitation and hypertensive response in hypertension^13^. Our study, along with others, has shown that AT1-R in the PVN induces mitochondria dysfunction and produces excessive amounts of ROS in peripheral angiotensin II (ANGII)-induced hypertension rats^14^,^15^. Glutamate is a well-known excitatory neurotransmitter, which participates in regulating neuronal excitation in the central nervous system (CNS). Neuronal activity in the PVN is regulated by glutamate and other excitatory neurotransmitters^16^,^17^. Previous studies show that oxidative stress contributes to modulating glutamatergic output in the PVN in hypertension rats^18^. These data suggest that RAS induces gene transcription of ROS, which leads to further glutamatergic output, and eventually to accelerated progression of hypertension.

PVN is a key site for the central control of sympathetic outflow and a predominant region for coordinating nervous system signals that regulate blood pressure, which plays a crucial role in renovascular hypertension^10^,^19^. Few studies on ExT in 2K1C hypertension models have focused on RAS, ROS, or glutamate within the PVN. Here, we test the hypothesis that ExT decreases blood pressure in renovascular hypertensive rats. Furthermore, we hypothesize that the favourable effect of exercise will be, in part, associated with RAS, ROS, and glutamate within the PVN of renovascular hypertensive rats.

## Methods

### Animal care

Experiments were performed in male Sprague-Dawley rats (eight weeks old and weighing 180–210 g). All rats were housed in a condition-controlled (21–23 °C, with the lights on from 7 pm to 7 am) room. They were permitted free access to standard rat chow and tap water. The rats were treated in accordance with the principles of the National Institutes of Health Guide for the Care and Use of Laboratory Animals (the US National Institutes of Health Publication No. 85–23, revised 1996). All protocols were approved by the Animal Care and Use Committee at Xi’an Jiaotong University.

### Renal artery clipping

Eight-week-old rats were anesthetized with xylazine (10 mg/kg) and ketamine (90 mg/kg) through intraperitoneal (i.p.) injection. Then, the rats were secured on the operating table, a right-flank incision was made in the abdomen, a silver clip (0.2 mm) was placed around the right renal artery, and then the flank incision was closed. Sham-clipped (Sham) rats underwent identical surgery without the silver clip. At the end of surgery, each rat received butorphanol tartrate (0.2 mg/kg subcutaneously) for an analgesic and penicillin for disinfection^19^,^20^.

### Exercise training

Four or five days after sham or renal artery clipping, the rats were randomly assigned to four groups: 2K1C + ExT group, 2K1C+ sedentary (Sed) group, SHAM + ExT group, SHAM + Sed group. The rats in ExT groups were assigned to eight weeks of exercise protocol (16 m/min, 50 min/d, and 5 d/wk).

### Measurement of mean arterial pressure (MAP)

Blood pressure was measured via a tail-cuff occlusion instrument and recording system, as described previously^21^. MAP data were averaged from 10 different measurements, which were collected either between 8 and 11 am or between 2 and 4 pm every week. After eight weeks ExT or Sed, rats were anaesthetized using a ketamine (90 mg/kg) and xylazine (10 mg/kg) mixture (i.p.). An incision along the blood vessels was made in the thigh near the groin, and the femoral artery was cannulated with polyethylene catheters to measure MAP. MAP readings were collected for 30 min and averaged. Subsequently, after the bilateral PVN microinjection of dizocilpine (MK801) or losartan (Los), MAP was measured again.

### Sympathetic neural recordings and PVN microinjections

Analysis of rectified and integrated renal sympathetic nerve activity (RSNA) and PVN microinjections were carried out described as previously^1^,^9^,^21^. Briefly, rats were anaesthetized using ketamine (90 mg/kg) and xylazine (10 mg/kg) and secured in a stereotaxic apparatus; then, bilateral PVN were implanted with cannulas and coordinates for the PVN were determined at 1.8 mm posterior, 0.4 mm lateral to the bregma, and 7.9 mm ventral to the zero level. The micropipette was filled with MK801 or Los. Continuous recordings of RSNA were taken at least 60 min after bilateral injection of MK801 or Los into the PVN.

### Collection of blood and tissue samples

At the end of the experiment, rats were anaesthetized for recording RSNA and MAP, and for collecting blood and brain tissue. Isolation of the PVN tissue from the brain using Palkovits’s microdissection procedure was performed as previously described^12^,^21^,^22^. Plasma and tissue samples were stored at a −80 °C for molecular and immunofluorescence analysis.

### Immunofluorescence staining

Rats were perfused with 300 ml phosphate-buffered saline solution (PBS, 0.01 M, pH 7.4) and 300 ml of 4% paraformaldehyde through the left ventricle. Cryostat was used to cut the PVN of rats into slices of about 14 μm. The primary antibodies used in these experiments were purchased from Santa Cruz Biotechnology, including gp^91phox^ (sc-20782, 1:200), p^47phox^ (sc-5827, 1:200), AT1-R (sc-1173, 1:200), and ACE (sc-20791, 1:200). Sections were imaged using a Nikon epifluorescence microscope.

Superoxide anion levels in PVN were determined by fluorescent-labelled dihydroethidium (DHE, Molecular Probes) staining. Brain sections (14 μm) were incubated with 1 mmol/L DHE at 37 °C for 10 min as previously described^1^. Sections were imaged using a Nikon epifluorescence microscope.

### Measurement of glutamate levels in the PVN

High-performance liquid chromatography with electrochemical detection (HPLC-EC) was used for measuring the level of glutamate in the PVN, as described previously^23^.

### Western blotting

Proteins extracted from punches of the PVN were used for analysing the expression of p^47phox^, AT1-R, and copper/zinc superoxide dismutase (Cu/Zn-SOD) by western blotting. Protein products were separated by 8% SDS-PAGE electrophoresis and transferred to nitrocellulose membranes. Blots were incubated with primary antibody overnight at 4 °C and secondary antibody (1:5000, Santa Cruz Biotechnology) for 1 h at room temperature. The following primary antibodies were purchased from Santa Cruz Biotechnology: p^47phox^ (sc-5827, 1:500), AT1-R (sc-1173, 1:500), and Cu/Zn-SOD (sc-11407, 1:500). Protein loading was controlled by probing all western blots with an anti-β-actin antibody (Santa Cruz Biotechnology) and normalizingp^47*phox*^, AT1-R, and Cu/Zn-SOD protein intensities to that of β-actin. Band densitieswere quantified using NIH’s Image J software.

### Biochemical assays

The level of ANG II in plasma was quantified using commercially available rat ELISA kits (Invitrogen) according to the manufacturer’s instructions.

### Statistical analysis

All data were analysed by ANOVA, followed by a post-hoc Tukey test. Blood pressure data were analysed by repeated measurements of ANOVA. All data are expressed as mean ± standard error (SE). The differences between mean values were considered to be statistically significant when the probability value of *P* was smaller than 0.05 (*P* < 0.05).

## Results

### Effects of ExT on MAP in renovascular hypertensive rats

**Figure 1:**
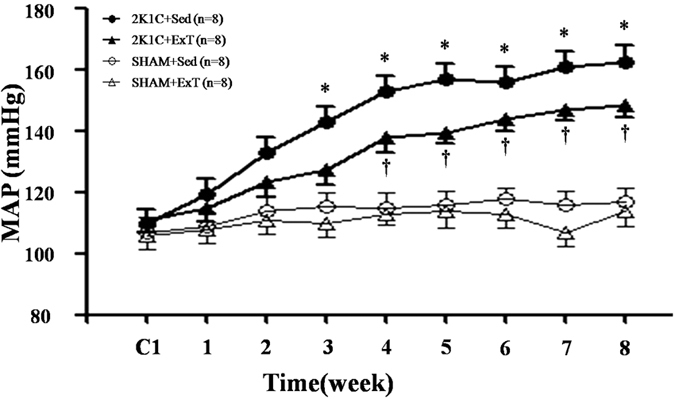
Effects of exercise training on mean arterial pressure in 2K1C rats. MAP was increased gradually after 2K1C-surgery compared with SHAM groups. ExT attenuated 2K1C-induced hypertension compared with 2K1C + Sed rats. MAP: mean arterial pressure; 2K1C: two-kidney, one-clip; ExT: exercise training; Sed: sedentary. Values are expressed as means ± SE. *P < 0.05 *vs* SHAM groups (SHAM + Sed or SHAM + ExT); ^†^*P* < 0.05 2K1C + ExT *vs* 2K1C + Sed.

2K1C hypertensive rats showed a significant increase in MAP compared with control rats. MAP remained elevated throughout the eight weeks of the study. Treatment with ExT reduced MAP in 2K1C-hypertensive rats (Fig. 1). However, ExT did not change MAP in SHAM + Sed and SHAM + ExT rats.

### Effects of PVN microinjection of MK801 or Los on MAP

PVN microinjection of MK801 or Los decreased MAP of 2K1C groups in hypertensive and ExT-hypertensive rats. This suggests that PVN microinjection of MK801or Los attenuates renovascular hypertension. Notably, PVN microinjection of Los exhibited lower MAP than PVN microinjection of MK801 in renovascular hypertensive rats (Table 1).

### Effects of eight weeks of ExT or PVN microinjection of MK801 or Los on renalsympathetic nerve activity (RSNA)

**Figure 2:**
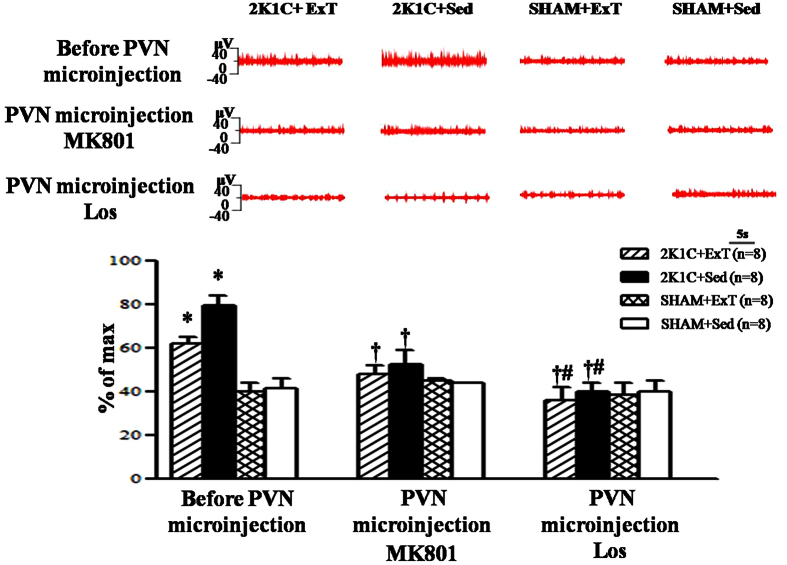
Effects of eight-week exercise training or PVN microinjection of MK801 or Los on RSNA in 2K1C rats and SHAM rats. RSNA was increased in 2K1C rats compared with SHAM rats. ExT attenuated RSNA compared with 2K1C + Sed rats. Treatment with PVN microinjection of MK801 or Los attenuated RSNA of 2K1C rats. PVN microinjection of Los exhibited lower RSNA (% of max) compared with PVN microinjection of MK801 in 2K1C rats. RSNA: renal sympathetic nerve activity; PVN: hypothalamic paraventricular nucleus; 2K1C: two-kidney, one-clip; ExT: exercise training; Sed: sedentary; MK801: dizocilpine; Los: losartan. Values are expressed as means ± SE. *P < 0.05 *vs* SHAM groups (SHAM + Sed or SHAM + ExT); ^†^*P* < 0.05 *vs* 2K1C groups (2K1C + ExT or 2K1C + Sed); ^#^*P* < 0.05 *vs* 2K1C+PVN microinjection of MK801 (2K1C + ExT+ PVN microinjection of MK801 or 2K1C + Sed + PVN microinjection of MK801.

RSNA was increased in 2K1C rats compared with that in SHAM rats. ExT treatment with PVNmicroinjection of MK801 or Los attenuated RSNA of 2K1C rats. PVN microinjection of Los exhibited lower RSNA (%of max) compared with PVN microinjection of MK801 in 2K1C rats (Fig. 2). These results suggest that ExT attenuates RSNA, in part, through decrease of glutamate in renovascular hypertensive rats.

### Effects of ExT on RAS in renovascular hypertensive rats

**Figure 3:**
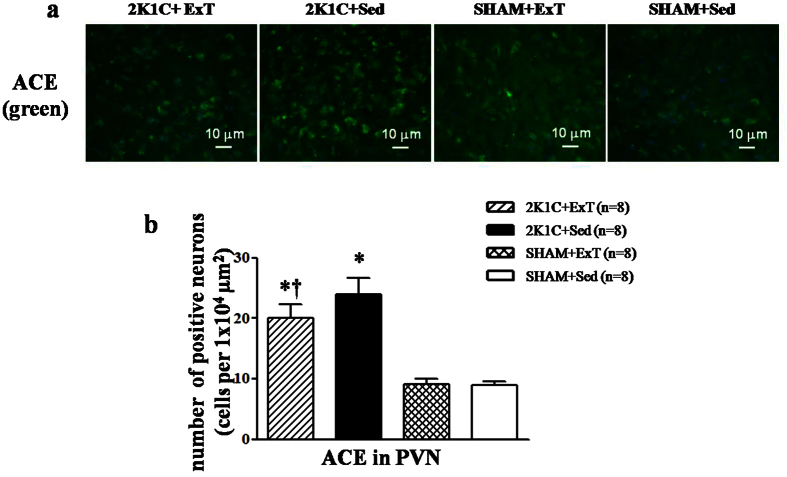
Effects of exercise training on the level of the ACE in the PVN in 2K1C rats by immunofluorescence staining. 2K1C rats had higher levels of ACE compared with SHAM rats. ExT decreased ACE expression in the PVN compared with 2K1C + Sed rats. (**a**) A representative immunofluorescence staining of ACE. (**b**) Densitometric analysis of immunofluorescent intensity of ACE. ACE: angiotensin-converting enzyme; PVN: hypothalamic paraventricular nucleus; 2K1C: two-kidney, one-clip; ExT: exercise training; Sed: sedentary. Values are expressed as means ± SE. *P < 0.05 *vs* SHAM groups (SHAM + Sed or SHAM + ExT); ^†^*P* < 0.05 2K1C + ExT *vs* 2K1C + Sed.

**Figure 4:**
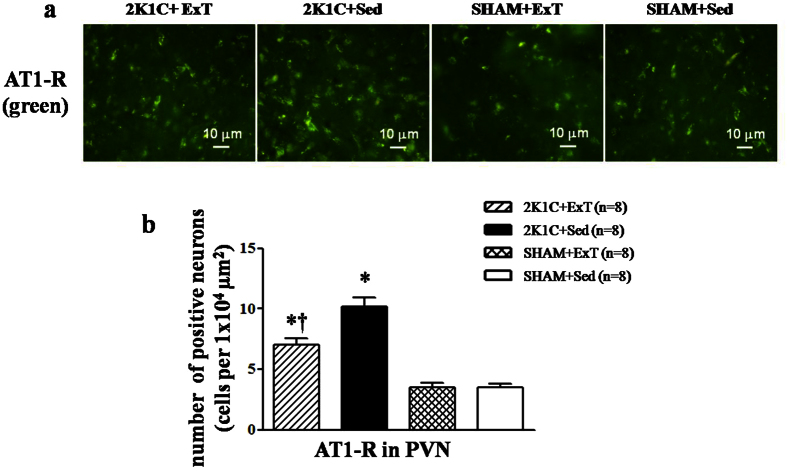
Effects of exercise training on the levels of the AT1-R in the PVN in 2K1C rats by immunofluorescence staining. 2K1C rats had higher levels of AT1-R compared with SHAM rats. ExT decreased AT1-R expression in the PVN compared with 2K1C + Sed rats. (**a**) A representative immunofluorescence staining of AT1-R. (**b**) Densitometric analysis of immunofluorescent intensity of AT1-R. AT1-R: angiotensin II type1 receptor; 2K1C: two-kidney, one-clip; PVN: hypothalamic paraventricular nucleus; ExT: exercise training; Sed: sedentary. Values are expressed as means ± SE. 2K1C *P < 0.05 *vs* SHAM groups (SHAM + Sed or SHAM + ExT); ^†^*P* < 0.05 2K1C + ExT *vs* 2K1C + Sed.

**Figure 5:**
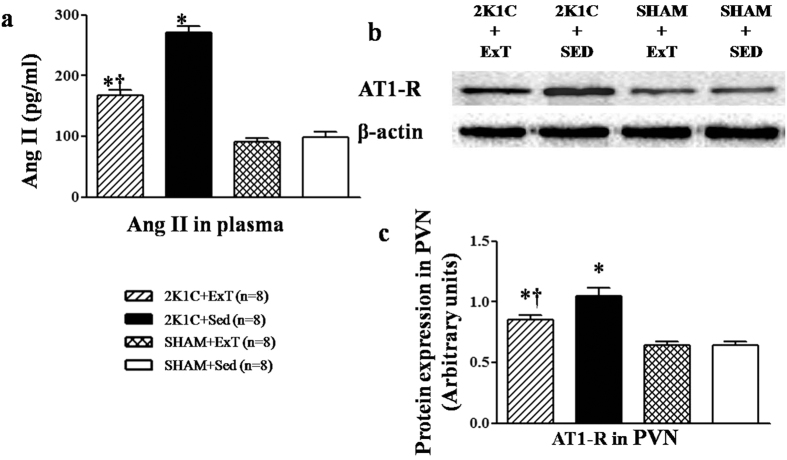
Effects of exercise training on the level of the AT1-R in the PVN and ANGII in the plasma in 2K1C rats. 2K1C rats had higher protein expression of AT1-R in the PVN and higher level of ANGII in the plasma compared with SHAM rats. ExT significantly decreased AT1-R expression in the PVN and ANGII in the plasma compared with 2K1C + Sed rats. AT1-R: angiotensin II type1 receptor; ANG II: angiotensin II; PVN: hypothalamic paraventricular nucleus; 2K1C: two-kidney, one-clip; ExT: exercise training; Sed: sedentary. Values are expressed as means ± SE. *P < 0.05 *vs* SHAM groups (SHAM + Sed or SHAM + ExT); ^†^*P* < 0.05 2K1C + ExT *vs* 2K1C + Sed. (**a**) Levels of ANGII in the plasma in different groups. (**b**) Representative immunoblot of AT1-R. (**c**) Densitometric analysis of protein expression of AT1-R.

Immunofluorescence revealed that renovascular hypertensive rats had a significant increase of ACE and AT1-R expression in the PVN compared with SHAM rats. ExT decreased ACE- and AT1-R-positive neurons in the PVN and decreased plasma levels of ANG II in renovascular hypertensive rats (Figs 3, 4 and 5a). ELISA analysis revealed that 2K1C rats had a higher level of ANG II in the plasma compared with SHAM rats (Fig. 5a). Western blotting showed that renovascular hypertension up-regulated the expression ofAT1-R compared with that in SHAM rats. ExT also attenuated the expression of AT1-R (Fig. 5b and 5c), suggesting that ExT attenuates RAS activation in renovascular hypertension.

### Effects of ExT on oxidative stress in the PVN of renovascular hypertensive rats

**Figure 6:**
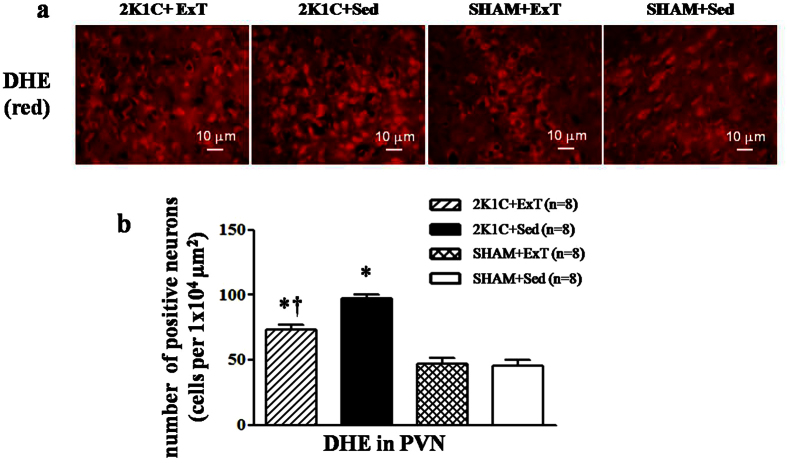
Effects of exercise training on the levels of the superoxide in the PVN in 2K1C rats. ROS activity was measured by fluorescent-labeled DHE staining. 2K1C rats had stronger fluorescence intensity labeled with DHE compared with SHAM rats. ExT significantly decreased DHE staining in the PVN of 2K1C rats. (**a**) A representative immunofluorescence image of DHE. (**b**) Densitometric analysis of immunofluorescent intensity of DHE in the PVN in different groups. DHE: dihydroethidium; PVN: hypothalamic paraventricular nucleus; 2K1C: two-kidney, one-clip; ExT: exercise training; Sed: sedentary. Values are expressed as means ± SE. *P < 0.05 *vs* SHAM groups (SHAM + Sed or SHAM + ExT); ^†^*P* < 0.05 2K1C + ExT *vs* 2K1C + Sed.

**Figure 7:**
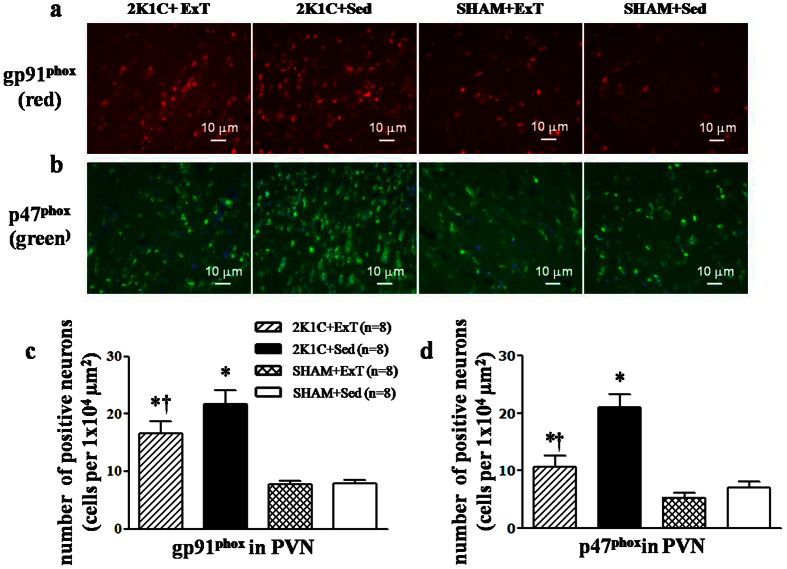
Effects of exercise training on gp^91phox^ and p^47phox^ in the PVN in 2K1C rats by immunofluorescence staining. 2K1C rats had higher levels of gp^91phox^ and p^47phox^ compared with SHAM rats. ExT decreased gp^91phox^ and p^47phox^ expression in the PVN compared with 2K1C + Sed rats. (**a**) A representative immunofluorescence staining of gp^91phox^. (**b**) A representative immunofluorescence staining of p^47phox^. (**c**) Densitometric analysis of immunofluorescent intensity of gp^91phox^ in the PVN in different groups. (**d**) Densitometric analysis of immunofluorescent intensity of p^47phox^ in the PVN in different groups. PVN: hypothalamic paraventricular nucleus; 2K1C: two-kidney, one-clip; ExT: exercise training; Sed: sedentary. Values are expressed as means ± SE. *P < 0.05 *vs* SHAM groups (SHAM + Sed or SHAM + ExT); ^†^*P* < 0.05 2K1C + ExT *vs* 2K1C + Sed.

**Figure 8:**
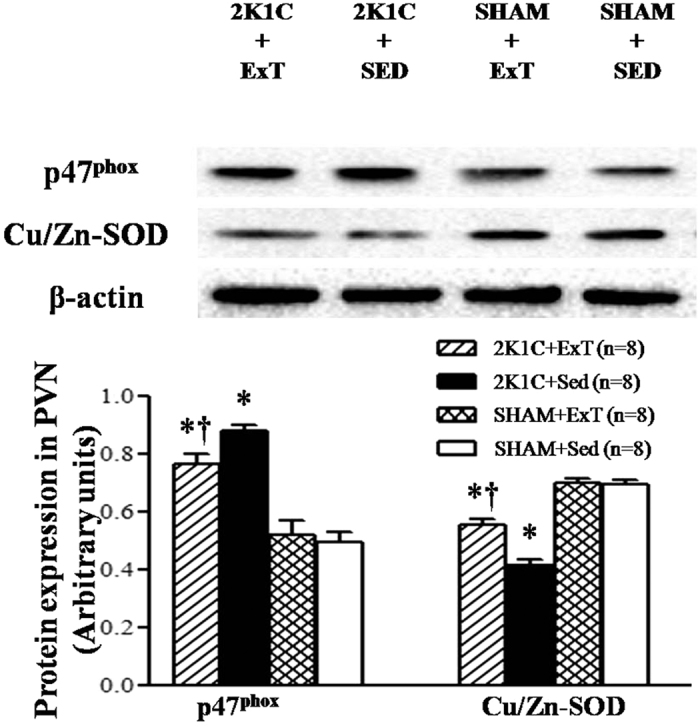
Effects of exercise training on the levels of p^47phox^ and Cu/Zn-SOD in the PVN in 2K1C rats by Western blotting. 2K1C rats had a lower level of Cu/Zn-SOD and a higher level of p^47phox^ compare with SHAM rats. ExT enhanced the expression of Cu/Zn-SOD and attenuated the expression of p^47phox^. (**a**) Representative immunoblot; (**b**) Densitometric analysis of protein expression of Cu/Zn-SOD and p^47phox^ in the PVN in different groups. Cu/Zn-SOD: copper/zinc superoxide dismutase; PVN: hypothalamic paraventricular nucleus; 2K1C: two-kidney, one-clip; ExT: exercise training; Sed: sedentary. Values are expressed as means ± SE. *P < 0.05 *vs* SHAM groups (SHAM+ Sed or SHAM + ExT); ^†^*P* < 0.05 2K1C + ExT *vs* 2K1C + Sed.

Immunofluorescence revealed that renovascular hypertensive rats had significant increase in the expression of p^47phox^, gp^91phox^, and DHE in the PVN compared with SHAM rats. ExT decreasedp^47phox^, gp^91phox^, and DHE-positive neurons in hypertensive rats (Fig. 6 and 7). Western blotting indicated that 2K1C hypertensive rats had lower levels of Cu/Zn-SOD and higher levels of p^47phox^ compared with SHAM rats. ExT enhanced the expression of Cu/Zn-SOD and decreased the expression of p^47phox^ (Fig. 8). This suggests that ExT attenuates oxidative stress in renovascular hypertension.

### Effects of ExT on glutamate in the PVN of renovascular hypertensive rats

**Figure 9:**
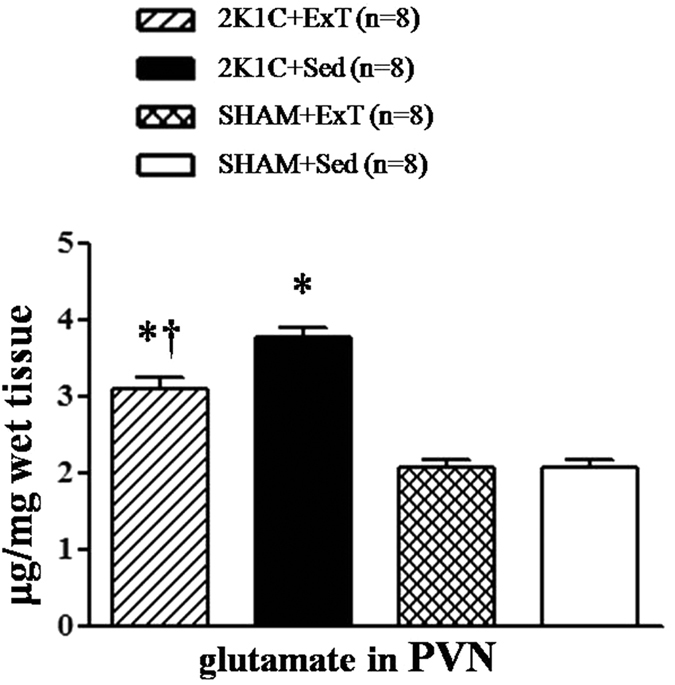
Effects of exercise training on the levels of glutamate in the PVN in 2K1C rats. 2K1C rats had higher levels of glutamate in the PVN compare with SHAM rats. ExT attenuated the level of glutamate compared with 2K1C + Sed rats. PVN: hypothalamic paraventricular nucleus; 2K1C: two-kidney, one-clip; ExT: exercise training; Sed: sedentary. Values are expressed as means ± SE. *P < 0.05 *vs* SHAM groups (SHAM + Sed or SHAM + ExT); ^†^*P* < 0.05 2K1C + ExT *vs* 2K1C + Sed.

We measured glutamate, a vital excitatory neurotransmitter, in the PVN by HPLC. These results revealed that 2K1C rats had higher levels of glutamate in the PVN compared with SHAM rats. This suggests that increased glutamate in the PVN contributes to elevated levels of blood pressure in renovascular hypertension. ExT attenuated the level of glutamate in the PVN compared with that in SHAM rats (Fig. 9). This suggests that ExT significantly decreases the level of glutamate in the PVN in renovascular hypertensive rats.

## Discussion

The novel findings of the present study are as follows: (1) ExT inhibited MAP and RSNA by attenuating ROS, RAS, and the excitatory neurotransmitter glutamate; (2) microinjection of MK801 or Los into the PVN decreased MAP and RSNA. We conclude that ExT decreases blood pressure in renovascular hypertensive rats, and this depressive favourable effect is associated with the RAS-ROS-glutamate pathway within the PVN.

Previous studies have reported that ExT is capable of decreasing blood pressure in hypertension and has been recommended as an effective nonpharmacological treatment for hypertension^23^,^24^,^25^,^26^. It also repored that neuronal plasticity plays an important role in the central regulation of ExT in hypertension^27^. Up-regulated glutamatergic outflow in the PVN contributes to an increase in blood pressure and sympathetic outflow in hypertensive rats^28^,^29^. In the present study, we confirmed that 2K1C rats have higher levels of glutamate in the PVN and that ExT significantly decreases the levels of glutamate in the PVN in renovascular hypertensive rats. This indicates that ExT is capable of attenuating the increased tonically active glutamatergic output in the PVN; however, the detailed mechanisms of ExT on glutamatergic levels in the PVN of renovascular hypertensive rats have not been firmly established.

One possibility is that ExT reduces oxidative stress in the PVN. It has been demonstrated that increased oxidative stress in the PVN contributes to sympathetic overactivityin hypertension^30^,^31^. Here, we found that ExT reduced the expression of p^47phox^ and gp^91*phox*^, but increased the expression of Cu/Zn-SOD in the PVN. We also showed that ExT reduced glutamatergic output in the PVN in renovascular hypertension by reducing oxidative stress.

In addition, our data suggest that ExT reduces glutamatergic output in the PVN in renovascular hypertension via reduction of oxidative stress, which is mediated by RAS. In a previous study, Li *et al*. found that renovascular hypertension is closely related to RAS activation in PVN^10^. AT1-R in the PVN induces over-production of ROS in rats with heart failure^32^. In our study, we proved that 2K1C rats had excessive oxidative stress and over-activated RAS in PVN. ExT significantly inhibited ACE, AT1-R, and glutamate in the PVN, and ANG II in plasma. ExT also attenuated ROS by augmenting Cu/Zn-SOD and decreasing p^47phox^ and gp^91phox^ in the PVN. However, some studies have reported that central PICs and nuclear factor-κB are also involved in the production of ROS^33^,^34^. In our research, we observed that microinjection of MK801 or Los into the PVN decreased MAP and RSNA in renovascular hypertensive rats. PVN microinjection of Los exhibited lower MAP and RSNA compared with that of MK801 in renovascular hypertensive rats. These results suggest that ExT attenuates hypertension, in part, through RAS-ROS-glutamate in renovascular hypertensive rats.

In summary, our data demonstrate that renovascular hypertension alters the RAS-ROS-glutamate pathway in the PVN and increases tonically active glutamatergic input in the PVN, which partly leads to an increase in MAP and RSNA. This indicates that ExT attenuates hypertension partly through the RAS-ROS-glutamate pathway in renovascular hypertensive rats. Our findings provide further evidence and insight into the beneficial effect of ExT on renovascular hypertension.

## Additional Information

**How to cite this article**: Zhang, Y. *et al*. Exercise training attenuates renovascular hypertension partly via RAS- ROS- glutamate pathway in the hypothalamic paraventricular nucleus. *Sci. Rep.* **6**, 37467; doi: 10.1038/srep37467 (2016).

**Publisher's note:** Springer Nature remains neutral with regard to jurisdictional claims in published maps and institutional affiliations.

## Figures and Tables

**Table 1:** MAP before and after PVN microinjection of MK801 or Los.

Group	Before PVN microinjection	PVN microinjection MK801	PVN microinjection Los
2KIC+ExT	139 ± 5	121 ± 6^*^	107 ± 9^*^
2KIC+Sed	157 ± 9	132 ± 11^†^	106 ± 7^†#^
SHAM+ExT	100 ± 7	102 ± 10	104 ± 9
SHAM+Sed	103 ± 7	104 ± 11	106 ± 8

Values are means ± SE. MAP, mean arterial pressure; ExT: exercise training; Sed: sedentary; PVN: hypothalamic paraventricular nucleus; MK801: dizocilpine; Los: losartan; *P < 0.05 *vs* 2K1C +ExT (before PVN microinjection); ^†^*P* < 0.05 *vs* 2K1C + Sed (before PVN microinjection); ^#^*P* < 0.05 2K1C + Sed (PVN microinjection MK801) *vs* 2K1C + Sed (PVN microinjection Los).
